# Emerging roles of small extracellular vesicles in metabolic reprogramming and drug resistance in cancers

**DOI:** 10.20517/cdr.2024.81

**Published:** 2024-09-27

**Authors:** Jingcun Shi, Ying Shen, Jianjun Zhang

**Affiliations:** ^1^Department of Oral and Maxillofacial-Head and Neck Oncology, Shanghai Ninth People’s Hospital, Shanghai Jiao Tong University School of Medicine; College of Stomatology, Shanghai Jiao Tong University; National Center for Stomatology; National Clinical Research Center for Oral Diseases; Shanghai Key Laboratory of Stomatology; Shanghai Research Institute of Stomatology; Shanghai Center of Head and Neck Oncology Clinical and Translational Science, Shanghai 200011, China.; ^2^Key Laboratory of Cell Differentiation and Apoptosis of Chinese Ministry of Education, Department of Pharmacology and Chemical Biology, Shanghai Jiao Tong University School of Medicine, Shanghai 200025, China.; ^3^Collaborative Innovation Center for Clinical and Translational Science by Chinese Ministry of Education & Shanghai, Shanghai 200025, China.

**Keywords:** Small extracellular vesicles, metabolic reprogramming, drug resistance, neoplasms, glycolysis, lipid metabolism, amino acid metabolism

## Abstract

Studies of carcinogenic metabolism have shown that cancer cells have significant metabolic adaptability and that their metabolic dynamics undergo extensive reprogramming, which is a fundamental feature of cancer. The Warburg effect describes the preference of cancer cells for glycolysis over oxidative phosphorylation (OXPHOS), even under aerobic conditions. However, metabolic reprogramming in cancer cells involves not only glycolysis but also changes in lipid and amino acid metabolism. The mechanisms of these metabolic shifts are critical for the discovery of novel cancer therapeutic targets. Despite advances in the field of oncology, chemotherapy resistance, including multidrug resistance, remains a challenge. Research has revealed a correlation between metabolic reprogramming and anticancer drug resistance, but the underlying complex mechanisms are not fully understood. In addition, small extracellular vesicles (sEVs) may play a role in expanding metabolic reprogramming and promoting the development of drug resistance by mediating intercellular communication. The aim of this review is to assess the metabolic reprogramming processes that intersect with resistance to anticancer therapy, with particular attention given to the changes in glycolysis, lipid metabolism, and amino acid metabolism that accompany this phenomenon. In addition, the role of sEVs in disseminating metabolic reprogramming and promoting the development of drug-resistant phenotypes will be critically evaluated.

## INTRODUCTION

Small extracellular vesicles (sEVs), commonly known as “exosomes”, are lipid bilayer-enclosed vesicles with diameters ranging from 30 to 160 nm^[1]^. These vesicles can sequester bioactive molecules, including proteins, nucleic acids, and lipids, thereby safeguarding them from degradation^[2]^. The biogenesis of sEVs involves a meticulously orchestrated biological sequence, commencing with the invagination of cellular membranes to form early endosomes. This process is subsequently followed by further invagination of endosomal membranes and culminates in the formation of intraluminal vesicles, which eventually form late endosomes or multivesicular bodies (MVBs). The cargo sorting within this process may either be contingent upon the endosomal sorting complex required for transport machinery or may proceed independently thereof^[3]^. In the usual course of events, MVBs coalesce with lysosomes, leading to their degradation. However, a subset of MVBs fuses with the plasma membrane, thereby releasing intraluminal vesicles into the extracellular milieu. During this transit and release, members of the small GTPase RAB family - Rab27a, Rab27b, Rab35, and Rab7 - in conjunction with the soluble N-ethylmaleimide-sensitive factor attachment protein receptor complex play key regulatory roles in the sorting and targeted transport of vesicles to secretory organelles^[4,5]^.

Numerous cell types, including cancer cells, have the capacity to generate and secrete sEVs. These sEVs facilitate intercellular communication by transporting specific biomolecules^[6]^. Increasing evidence has suggested that within the context of the tumor microenvironment (TME), neoplastic cells engage in interactions with neighboring cells via the release of sEVs. These interactions are key in the modulation of various facets of tumor biology, including tumor proliferation, progression, angiogenesis, and immune evasion, and increase tumor aggressiveness and metastatic potential^[7,8]^. Recent investigations have focused primarily on elucidating the impact of sEVs on the biological attributes of tumors and immune cells. However, emerging data indicate that the contents of sEVs may also influence the metabolic status of recipient cells and potentially contribute to the propagation of chemotherapeutic drug resistance^[9-11]^. Nevertheless, the precise mechanisms underlying the sEVs-mediated transmission of chemotherapy resistance remain incompletely understood.

In recent years, the examination of oncogenic metabolism has garnered widespread interest within the scientific community. Neoplastic cells demonstrate a remarkable capacity for metabolic adaptation in nutrient-depleted environments, procuring essential nutrients to facilitate tumoral expansion. In contrast to those of nonmalignant cells, the metabolic dynamics of cancer cells are subject to extensive reprogramming, which supports the acquisition and sustenance of malignant traits. Consequently, such metabolic reprogramming is increasingly recognized as a fundamental hallmark of cancer^[12]^. Otto Warburg was the pioneer in defining metabolic dysregulation in cancer cells, noting their greater propensity for glucose uptake than their nontransformed counterparts. Additionally, he reported that glycolytic activity in cancer cells predominates over oxidative phosphorylation (OXPHOS), even in the presence of ample oxygen, and this phenomenon has become known as the Warburg effect^[13]^. Although glycolysis is less efficient than OXPHOS in energy conversion, it enables more rapid production of adenosine triphosphate (ATP) and fosters the biosynthesis of nucleotides, amino acids, nicotinamide adenine dinucleotide phosphate (NADPH), and other biomolecules required for cellular proliferation^[14]^. Subsequent investigations revealed that the phenomenon of metabolic reprogramming in cancer extends beyond the Warburg effect, as it includes extensive alterations in glucose, amino acid, and lipid metabolism^[15,16]^. Through the exploration of metabolic reprogramming in neoplastic contexts, the molecular mechanisms governing these metabolic shifts could be elucidated, and potential therapeutic targets for cancer treatment could be identified.

Recent advancements in oncology have significantly expanded the spectrum and accessibility of pharmaceutical interventions for neoplastic diseases. Despite this progress, the incidence of chemotherapeutic resistance has increased, and multidrug resistance poses a formidable challenge to the efficacy of antineoplastic regimens^[17,18]^. Chemotherapeutics are designed to suppress the proliferation of heterogeneous tumor cell populations by exerting environmental stress. However, a subpopulation of cells may evolve mechanisms to circumvent therapeutic pressures, thereby diminishing their susceptibility to these agents. A multifaceted array of processes contributes to the development of therapeutic tolerance within tumors. For example, malignant cells can actively remove cytotoxic compounds through increased expression of ATP-binding cassette (ABC) transporters, effectively sequestering these agents away from intracellular targets^[19,20]^. Furthermore, enhanced capacities for DNA damage recognition and repair, induction of epithelial-to-mesenchymal transitions, alterations in drug target sequences, dysregulation of epigenetic landscapes, and perturbations in microRNA (miRNA) profiles have all been implicated in fostering a state of treatment resilience^[21,22]^. Recent studies have revealed a significant correlation between the reprogramming of neoplastic metabolism and the acquisition of resistance to chemotherapeutics^[23,24]^. However, the intricate underlying mechanisms regulating this relationship remain elusive. This review aims to thoroughly assess the metabolic reprogramming processes related to the development of cancer resistance, with a particular emphasis on the changes in glycolysis, lipid metabolism, and amino acid metabolism that accompany this phenomenon. Additionally, the role of sEVs in promoting metabolic reprogramming and facilitating the development of drug-resistant phenotypes will be critically evaluated.

## METABOLIC REPROGRAMMING REGULATES SENSITIVITY TO ANTITUMOR THERAPY

### Reprogramming of glucose metabolism

Alterations in glucose utilization significantly alter the rate of glycolysis, a key metabolic pathway implicated in the development of chemotherapeutic resistance^[25,26]^. Compelling evidence has shown that glucose activates the cyclic GMP-AMP synthase/stimulator of interferon gene (cGAS/STING) signaling axis by preserving the expression of TREX2. This in turn stimulates NSUN2, which promotes tumorigenesis and fosters resistance to immunotherapy^[27]^. An increase in glucose metabolic flux concurrently increases cardiolipin synthesis via increased glycerolipid biosynthesis. The resulting accumulation of cardiolipin decreases radiation-induced apoptosis through the inhibition of cytochrome c release, conferring a survival advantage to neoplastic cells. Central to these metabolic adaptations is the mTORC1/hypoxia-inducible factor-1α (HIF-1α)/SREBP1 signaling pathway, which orchestrates this metabolic reprogramming. Targeted interventions aimed at mTORC1 or the cardiolipin synthetic pathway may thus represent a strategy to sensitize tumors to radiation therapy^[28]^. Conversely, the emulation of glucose deprivation through the use of glutaminase (GLS) inhibitors increases the susceptibility of intrahepatic cholangiocarcinoma to chemotherapy, indicating the potential for metabolic modulation as an adjunct to conventional anticancer regimens^[16]^. Notably, a recent report by Park *et al.* reported that glucose deprivation triggers compensatory activation of the glycolytic pathway mediated by ELAVL2/4, thereby increasing tumor resistance to chemotherapy^[29]^. These findings indicate that glycolytic metabolism activation may increase chemotherapy resistance or radiotherapy resistance to tumor therapy.

Emerging evidence from recent investigations has elucidated the pivotal role of glucose transporters (GLUTs) and glycolytic enzymes in conferring resistance to chemotherapy^[30]^. Notably, ALKBH5, an N6-methyladenosine (m6A) demethylase, is significantly upregulated in breast cancer cells resistant to HER2-targeted therapies. This study revealed that ALKBH5 increases glycolysis in drug-resistant breast cancer cells by promoting m6A demethylation of GLUT4 messenger RNA (mRNA), thereby increasing GLUT4 expression^[31]^. Lactate dehydrogenase A (LDHA) is a crucial enzyme involved in both glycolysis and gluconeogenesis that plays a fundamental role in modulating tumor resistance to pharmacological interventions^[32]^. Acylphosphatase 1 (ACYP1) interacts with HSP90 to regulate the expression and stability of the oncogene cellular Myc (c-Myc). ACYP1 exacerbates the Warburg effect through activation of the Myc/LDHA axis, contributing to its tumor-supportive effects. Combinatorial targeting of ACYP1 alongside lenvatinib has been demonstrated to substantially mitigate lenvatinib resistance and impede tumor progression^[33]^. Furthermore, the circular RNA (circRNA) ARHGAP29 has been identified as a molecule capable of augmenting LDHA expression via its interaction with insulin-like growth factor 2 mRNA-binding protein 2 and c-Myc^[34]^. The long noncoding RNA (lncRNA) DIO3OS preserves the integrity of the LDHA 3’ untranslated region (3’UTR) and upregulates LDHA expression through its interaction with PTBP1, thereby stimulating glycolysis in drug-resistant breast cancer cells^[35]^. Additional glycolytic enzymes, including hexokinase (HK)^[36-38]^, 6-phosphofructo-2-kinase/fructose-2,6-bisphosphatase^[39,40]^, fructose diphosphate aldolase^[41,42]^, phosphoglycerate kinase (PGK)^[43,44]^, and pyruvate kinase (PK)^[45-47]^, have been implicated in the development of antineoplastic drug resistance, indicating the importance of glycolytic reprogramming in the evolution of treatment-refractory cancers.

**Figure 1:**
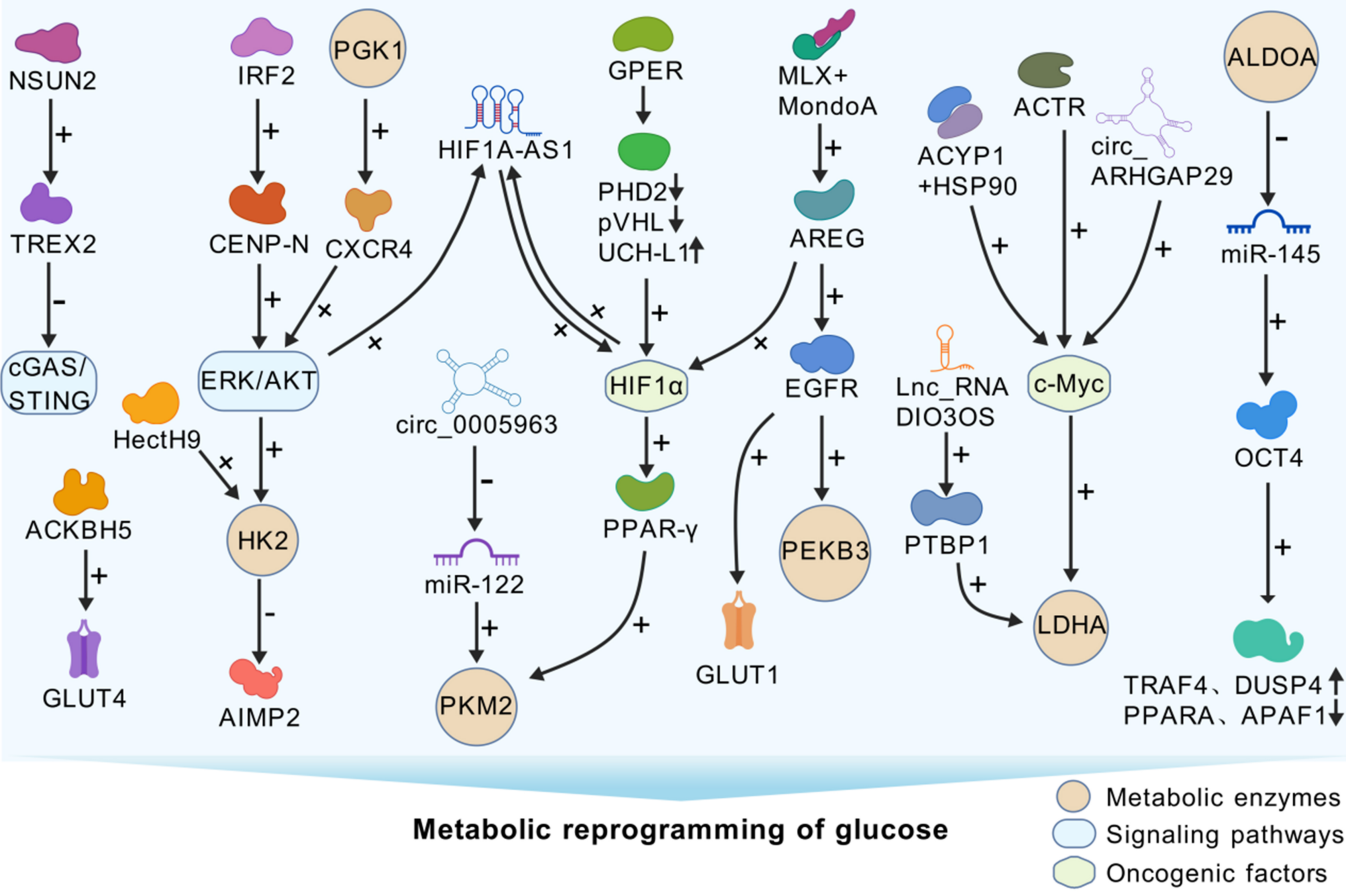
Glucose metabolic reprogramming regulates sensitivity to antitumor therapy. cGAS/STING: Cyclic GMP-AMP synthase/stimulator of interferon gene; ERK/AKT: extracellular regulated protein kinases/protein kinase B; HK2: hexokinase 2; PGK1: phosphoglycerate kinase 1; PKM2: pyruvate kinase M2; HIF-1α: hypoxia-inducible factor-1α; PEKB3: phosphofructokinase-2/fructose-2,6-biphosphatase 3; LDHA: lactate dehydrogenase A; c-Myc: cellular Myc; ALODA: aldolase A.

The activation of specific signaling pathways and oncogenic transcription factors has been shown to modulate glucose metabolism and confer drug resistance in cancerous cells. Thus, the PI3K-protein kinase B (AKT) pathway and key transcription factors, such as HIF-1α and c-Myc, play crucial roles^[48-54]^. PGK1 has been implicated in the proliferation of renal clear cell carcinoma and the development of sorafenib resistance, facilitated by the acceleration of glycolysis and the concomitant activation of the CXCR4/extracellular regulated protein kinase (ERK) signaling axis^[55]^. AKT increases glucose uptake by increasing the amount of GLUT1 and GLUT4 transporters in the membrane. Furthermore, AKT contributes to the phosphorylation of HK-2, thereby stimulating its translocation to the mitochondria. The lncRNA HIF1A-AS1 promotes the interaction between AKT and YB1, which in turn increases the translation of HIF1α. Additionally, HIF1α can directly engage with the HIF1α response element within the HIF1A-AS1 promoter region, thereby increasing HIF1A-AS1 transcription. This cyclic positive feedback mechanism between the two entities amplifies glycolysis and increases resistance to gemcitabine^[56]^. In nasopharyngeal carcinoma cells, CENP-N forms a complex with AKT, impacting tumor cell glucose metabolism and promoting malignant progression^[57]^. The AKT inhibitor afuresertib, when used in conjunction with carboplatin and paclitaxel, exhibited promising outcomes in a phase I clinical trial for the treatment of recurrent platinum-resistant ovarian cancer^[58]^. These findings collectively suggest that the reprogramming of glycolysis mediated by oncogenic transcription factors or signaling pathways increases tumor cell survival and promotes the progression of cancer [Figure 1].

Lactic acid is a product of glycolysis and has been increasingly recognized for its role in the development of drug resistance. Tumor cells exhibit increased rates of glucose uptake and lactate secretion, even under oxygen-replete conditions, a phenomenon referred to as aerobic glycolysis or the Warburg effect^[59]^. Notably, the accumulation of high concentrations of lactic acid not only remodels the TME but also serves as an alternative metabolic substrate for cancer cells, contributing to immunosuppression and therapeutic resistance^[60]^. Comparative analyses revealed that compared with their parental MCF-7 counterparts, tamoxifen-resistant MCF-7 cells exhibit increased levels of glycolytic enzymes and increased tolerance to growth environments with elevated lactic acid levels. Monocarboxylic acid transporter 1 (MCT1) and LDHB are key mediators that facilitate the influx of lactic acid and its conversion back to pyruvate, respectively^[61]^. Additionally, Feng *et al.* reported that the glycolytic enzyme phosphoglycerate mutase 1 contributes to paclitaxel resistance by facilitating the production of pyruvate and/or lactic acid^[62]^. These findings indicate the critical influence of dysregulated glycolysis and the associated accumulation of lactic acid in the development of a resistant tumor phenotype, highlighting potential vulnerabilities for targeted intervention in cancer therapy.

### Lipid metabolism reprogramming

The reprogramming of lipid metabolism is frequently observed in aggressive tumors and is closely related to both the responsiveness to and tolerance of antitumor therapies^[15]^. The de novo synthesis of lipids confers resistance to tumor cells, facilitating their growth and survival through various mechanisms. Evidence suggests that the metabolic shift of ovarian cancer cells from glycolytic dependency to a reliance on fatty acid (FA) metabolism increases their capacity to endure the oxidative stress induced by cisplatin^[63]^. Fatty acid synthase (FASN) is an integral enzyme in de novo FA synthesis. It is upregulated in drug-resistant tumor cells and contributes to therapeutic resistance by modulating the polyunsaturation of membrane lipids^[64-71]^. Central adipose-derived transcription factors play pivotal roles in regulating genes involved in cholesterol and FA metabolism. Inhibitors that target central adipose-derived transcription factors have been shown to increase lipid peroxidation and reverse drug resistance in melanoma cells^[72]^. Furthermore, targeting acetyl-CoA carboxylase-1 has demonstrated efficacy in curtailing tumor growth within patient-derived xenografts exhibiting resistance^[73]^. Additionally, G protein-coupled receptor 120 (GPR120) increases the synthesis of FA, which activates GPR120 signaling through positive feedback. This upregulation of GPR120 via the AKT/NF-κB pathway increases the expression of the ABC transporter, which reduces the intracellular concentrations of chemotherapeutic agents, culminating in chemoresistance^[74]^. Arachidonic acid (ARA), released from membrane phospholipids, is metabolized to the active metabolite eicosanoid acid by two rate-limiting enzymes through the action of cytoplasmic phospholipase A2α (cPLA2α). Cyclooxygenase (COX) generates prostaglandins (PG), prostacyclins, and thromboxanes, while lipoxygenase (LOX) catalyzes the production of leukotrienes (LT) and hydroxy-eicosatetraenoic acid (HETE)^[75]^. Once secreted, these compounds act in either an autocrine or paracrine fashion on the producing cells or adjacent cells, respectively, thereby mediating tumor promotion and progression^[76-83]^. Moreover, elevated cholesterol levels within lipid rafts have been shown to diminish the inhibitory effect of gefitinib on EGFR tyrosine kinases, thereby causing chemoresistance^[84]^. Notably, when tumors are in the early stages and more reliant on cholesterol for sustaining oncogenic signaling, statins can effectively curb cancer initiation and proliferation by inhibiting cholesterol synthesis^[85]^. In summary, the abnormal synthesis of novel FAs and cholesterol provides tumor cells with a continuous supply of membrane precursors, signaling molecules, and energy substrates, enabling rapid tumor growth even under conditions of nutrient limitation and hypoxia. The reprogramming of lipid anabolic pathways alters the responsiveness of tumors to treatment, ultimately culminating in drug resistance.

Lipid uptake and oxidative metabolism are pivotal in the development of drug resistance among tumors^[86]^. Similar to glycolysis, the absorption of exogenous FAs is facilitated by dedicated transporters, with notable examples including FA translocases, FA transport proteins, and FA binding proteins^[87-89]^. In a prostate cancer-prone Pten^-/-^ mouse model, FA translocase has been shown to facilitate FA uptake and storage, significantly impacting fatty acid oxidative (FAO) metabolism and reversing increases in acylcarnitines, monoacylglycerols, and phospholipid hydrolysates induced by Pten deficiency^[90]^. Alicea *et al.* reported that inhibiting FA transport protein 2 diminishes lipid uptake and mitochondrial function, effectively restoring melanoma cell sensitivity to BRAF/MEK inhibitors^[91]^. Carnitine palmitoyl transferase (CPT) I and II are rate-limiting enzymes for mitochondrial FA transport and play key roles in FAO^[92-96]^. Inhibition of FAO by etomoxir or genetic ablation of CPT1A/CPT2 markedly inhibited the ERK1/2 pathway and increased the responsiveness of breast cancer cells to radiotherapy^[97]^. Peroxisome proliferator-activated receptor γ is a transcription factor that governs genes related to lipid metabolism and is thought to promote FAO upon activation, thereby inducing chemoresistance^[98]^. Adipocytes neighboring tumor sites can protect cancer cells from antineoplastic agents by increasing FAO and secreting soluble factors that modulate the sensitivity of HER2-positive breast cancer cells to lapatinib^[99]^. Triacylglycerol (TAG) serves as the primary storage form for excess intracellular FAs within lipid droplets (LDs)^[100]^. These TAGs undergo hydrolysis and decomposition through a sequence of three cytoplasmic lipase-mediated reactions, known as neutral lipolysis, yielding FAs and glycerol^[101]^. Research has indicated that inhibiting this metabolic pathway can impact metastasis formation, either by directly targeting lipase-mediated enzymes such as monoacylglycerol lipase (MAGL) and hormone-sensitive lipase (HSL), or indirectly by affecting long-chain acyl-CoA synthetases (ACSL), which facilitate the activation of long-chain FAs^[102-104]^. Moreover, the development of resistance to anticancer therapies is closely linked to the assimilation of exogenous cholesterol. Cisplatin-resistant ovarian cancer cells exhibit decreased expression of farnesyl diphosphate synthase and OSC and increased expression of low-density lipoprotein receptors, indicating a reduction in cholesterol biosynthesis and a concomitant increase in extracellular cholesterol uptake. Notably, lipid deprivation has been shown to increase the sensitivity of resistant cells to cisplatin^[105]^. Targeting the transporters and key enzymes involved in lipid uptake and oxidative consumption is a potential strategy for restoring the therapeutic sensitivity of cancer cells.

Lipid storage significantly impacts antitumor treatment efficacy and disease prognosis. Increased intake of exogenous FAs leads to increased FA storage in LDs^[106,107]^. LDs sequester excess FAs in the form of TAG and sterol esters. In response to the lipid toxicity and lipid peroxidation induced by anticancer therapy, LDs can isolate excess free FAs and maintain lipid homeostasis. LDs increase the aggressiveness and drug resistance of tumor cells by mitigating cellular stress^[108-112]^. Indeed, the levels of LDs and their colocalization with mitochondria are significantly greater in chemotherapy-resistant breast cancer cell lines than in parental cells^[113]^. In an ovarian cancer xenotransplantation model treated with bevacizumab, lipid metabolism was upregulated, and LD accumulation was increased. Inhibiting the uptake of exogenous lipids reduces LD accumulation and enhances the antitumor effect of bevacizumab^[114]^. Stearoyl-CoA desaturase 1 (SCD1) is a key enzyme in the synthesis of monounsaturated FAs. In non-small cell lung cancer (NSCLC) cell lines susceptible to EGFR mutations, SCD1 expression is elevated, thereby increasing the intracellular LD content. Additionally, oleic acid, the enzymatic product of SCD1, can inhibit the cytotoxic effects of gefitinib and osimertinib in EGFR-activated mutant cell lines. Inhibitors of lipid metabolism can reverse these biological effects and increase the sensitivity of NSCLC cell lines to gefitinib^[115,116]^. In summary, lipid metabolism and lipid storage in LDs are not only adaptive mechanisms for tumor cells to cope with therapeutic stress but also potential therapeutic targets, and their modulation may be of great value for improving the efficacy of antitumor therapy and disease prognosis.

**Figure 2:**
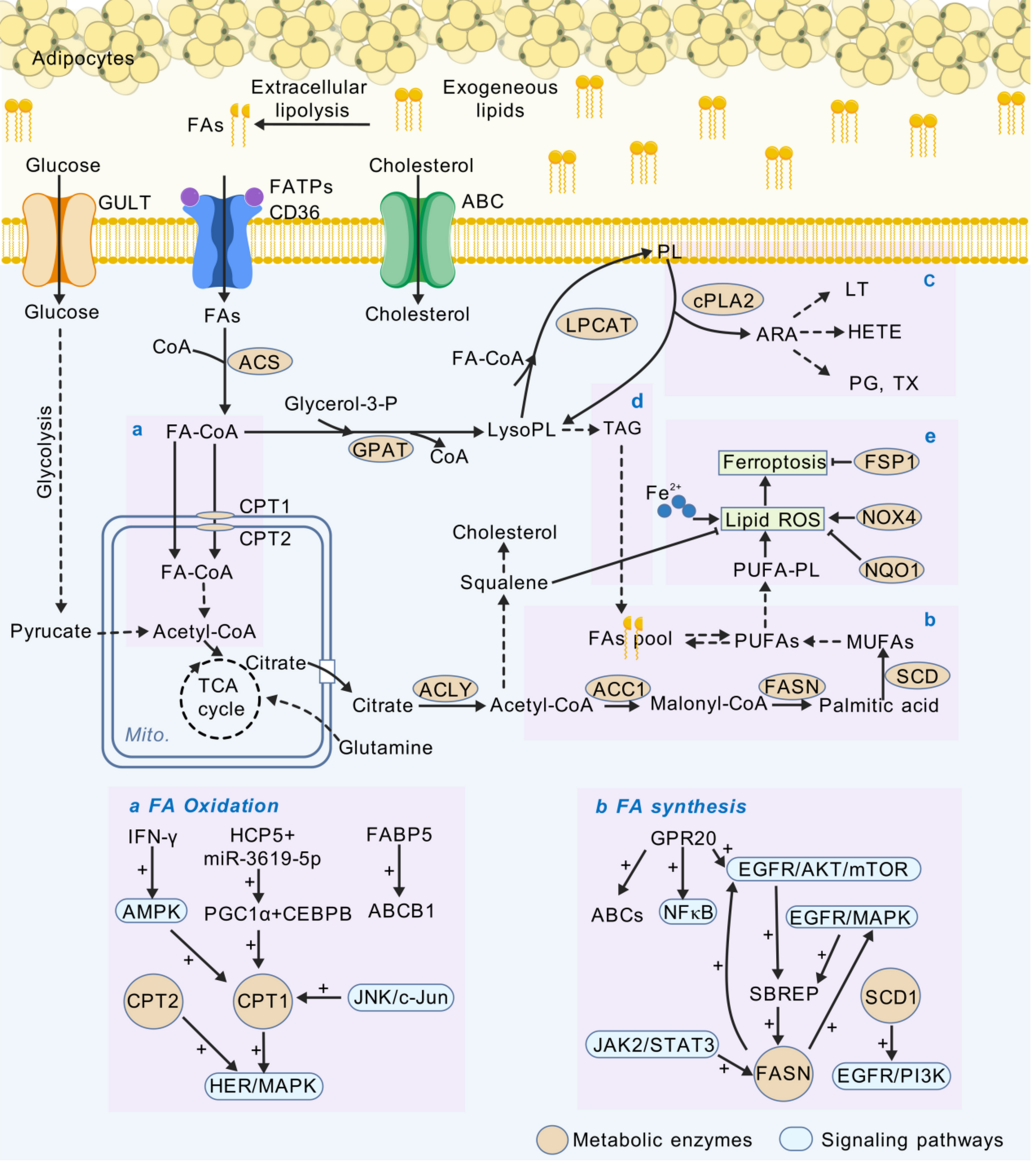
Lipid metabolic reprogramming regulates sensitivity to antitumor therapy. a: Fatty acid β-oxidation. b: De novo lipogenesis. c: Eicosanoid synthesis. d: Neutral lipolysis. e: Lipid ROS and ferroptosis. ACLY: ATP-citrate lyase; ACC1: acetyl-CoA carboxylase; FASN: fatty acid synthase; SCD: stearoyl-CoA desaturase; AMPK: adenosine 5’-monophosphate (AMP)-activated protein kinase; CPT: carnitine palmitoyl transferase; JNK: c-Jun N-terminal kinase; HER: human epidermal growth factor receptor; MAPK: mitogen-activated protein kinase; NF-κB: nuclear factor kappa-light-chain-enhancer of activated B cells; EGFR: epidermal growth factor receptor; AKT: protein kinase B; JAK2/STAT3: Janus kinase 2/signal transducer and activator of transcription 3; PI3K: phosphatidylinositol 3-kinase.

Recent investigations have elucidated the role of lipid metabolism in the progression of malignant tumors, particularly through its regulation of ferroptosis, an iron-dependent, nonapoptotic form of cell death primarily driven by excessive lipid peroxidation within cellular membranes^[117]^. In human pancreatic ductal adenocarcinoma (PDAC) cells, pyruvate dehydrogenase kinase 4 inhibits FA peroxidation by restricting pyruvate oxidation and FA synthesis, thereby preventing ferroptosis^[118]^. Lee *et al.* reported that sequestration of excess polyunsaturated FAs such as TAG within LDs during cell cycle arrest leads to the inhibition of ferroptosis^[119]^. Furthermore, SCD1-mediated FA desaturation and FA-binding protein-4-mediated LD biogenesis play pivotal roles in circumventing oxidative stress-induced ferroptosis in tumor cells^[120]^. Recently, the induction of ferroptosis in tumor cells has emerged as a promising anticancer strategy^[121]^. Luo *et al.* successfully reversed the ferroptosis resistance induced by the deletion of long-chain ACSL4 through the targeted delivery of ferroptotic lipids, such as arachidonic acylphosphatidyl ethanolamine^[122]^. Therefore, a more comprehensive understanding of the molecular mechanisms underlying dysregulated lipid metabolism and ferroptosis may reveal novel approaches for preventing resistance to cancer treatment [Figure 2].

### Amino acid metabolic reprogramming

**Figure 3:**
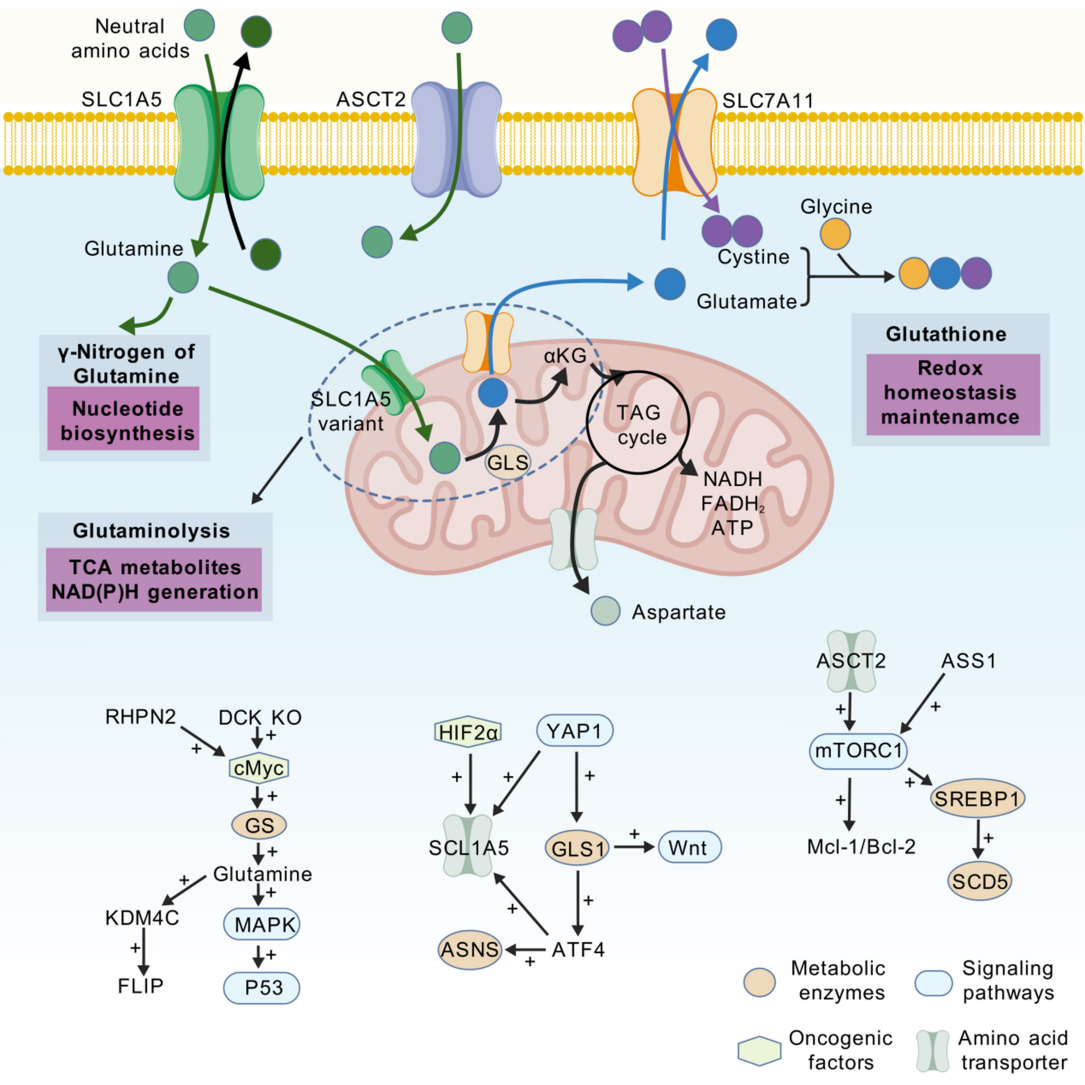
Glutamine metabolic reprogramming regulates sensitivity to antitumor therapy. SLC1A5: Solute carrier family 1 member 5; ASCT2: alanine, serine, cysteine-preferring transporter 2; SLC7A11: solute carrier family 7 member 11; GLS: glutaminase; cMyc: cellular Myc; GS: glutamine synthetase; MAPK: mitogen-activated protein kinase; P53: tumor protein p53; HIF2α: hypoxia-inducible factor 2 alpha; YAP1: yes-associated protein 1; Wnt: wingless-related integration site; ASNS: asparagine synthetase; mTORC1: mechanistic target of rapamycin complex 1; SREBP1: sterol regulatory element-binding protein-1; SCD5: stearoyl-coenzyme A desaturase 5.

Amino acid metabolism is integral to cell biomass production, energy generation, and the maintenance of redox homeostasis. Dysregulation of amino acid metabolism within tumor cells supports their metabolic needs and aids in coping with therapeutically induced stress^[123]^. Metabolic reprogramming of glutamine is a frequent occurrence in cancer and ranks second only to glycolysis in its significance [Figure 3]^[124-132]^. Ying *et al.* reported that transcriptome-based glutamine metabolism scores serve as robust prognostic indicators and are closely correlated with overall survival, responsiveness to immunotherapy, and the extent of immune cell infiltration^[133]^. Glutamine metabolism is closely related to nucleotide biosynthesis. In cells that are resistant to radiation, glycolysis, mitochondrial OXPHOS, and tricarboxylic acid cycle activity are reduced, and the capacity for glutamine assimilation is increased. Notably, glutamine synthetase promotes radioresistance by facilitating DNA repair and nucleotide metabolism^[134]^. Furthermore, nutrient deprivation has been shown to significantly disrupt the processing of precursor ribosomal RNA, leading to the accumulation of immature rRNA. Following amino acid deprivation, replenishment with glutamine alone can activate the p53 pathway, causing tumor cell apoptosis^[135]^. In addition to nucleotide biosynthesis, glutamine metabolism plays a pivotal role in regulating redox balance. In head and neck squamous cell carcinoma cells, the uptake of glutamine and the activity of glutamate dehydrogenase are inhibited by sulfapyridine, promoting mitochondrial metabolism and increasing the levels of reactive oxygen species (ROS), which culminate in oxidative damage^[136]^. The expression of the SLC1A5 variant, under the regulation of HIF-2α, promotes glutamine transport into the mitochondria, a process that increases ATP production and glutathione synthesis, which leads to gemcitabine resistance in pancreatic cancer cells^[137]^. Additionally, increased GLS1 activity has been demonstrated to increase redox signaling in hepatocellular carcinoma (HCC). Moreover, glutamine deprivation or treatment with GLS inhibitors may impede tumor progression by increasing intracellular ROS levels^[138]^. Glutamine metabolism also governs oxidative metabolic processes. Hu *et al.* reported that ASS1 induces erastin resistance in NSCLC cells via activation of the mTORC1-SREBP1-SCD5 pathway, promoting the reductive carboxylation of glutamine^[139]^. Notably, dietary intake of glutamine has proven effective in slowing melanoma growth, prolonging survival, and increasing responsiveness to BRAF inhibitor therapy. Elevated concentrations of glutamine and its downstream metabolite, alpha-ketoglutaric acid, within tumors lead to histone H3K4me3 hypomethylation, thereby inhibiting the activation of oncogenic pathways^[140,141]^. In summary, the dysregulation of glutamine metabolism within tumor cells confers therapeutic resistance by modulating nucleotide synthesis, ROS production, and ATP production.

Accumulating evidence suggests that regulating methionine metabolism to maintain equilibrium in nucleotide pools and redox states within cancerous and immune cells could resensitize tumors to chemotherapeutic agents. Methionine plays a pivotal role as a component of the folate cycle and provides the necessary precursor substances for the biosynthesis of purines and pyrimidines. In colorectal cancer patient-derived xenograft models characterized by RAS mutations, restricting methionine intake increases tumor cell susceptibility to 5-fluorouracil^[142]^. This increased sensitivity is proposed to result from the increased consumption of homocysteine and 5,10-methylene tetrahydrofolate by tumor cells, a process that inhibits folate cycling and nucleotide synthesis^[143]^. Single-carbon metabolism provides methyl groups for cellular methylation reactions. Stem cells are dependent on MAT2A enzymes to catalyze the synthesis of the methyl donor S-adenosylmethionine from methionine, a process essential for the maintenance of epigenomic stability. Investigations have revealed that methionine metabolism and MAT2A-mediated methylation are significantly increased in tumor-initiating cells, resulting in a reliance on exogenous methionine^[144]^. Methionine deprivation impedes cancer stem cells (CSCs) by reducing S-adenosylmethionine levels. The combination of methionine depletion with MAT2A inhibition represents a promising therapeutic strategy for targeting drug-resistant CSCs^[145]^.

Aspartic acid serves as a precursor for the tricarboxylic acid cycle, aids in maintaining the redox equilibrium of NAD+/NADH and contributes to nucleotide biosynthesis. Its role becomes critical when the electron transport chain is compromised, as it supports cell proliferation and significantly correlates with tumor cell resistance to pharmacological agents. In estrogen receptor (ER)-positive breast cancer cells, endocrine therapy resistance has been linked to increased activity of the SLC1A2 transporter. This facilitates the uptake of acidic amino acids, leading to elevated intracellular levels of aspartate and glutamate^[146]^. L-asparaginase (ASNase) is a cornerstone in the treatment of acute lymphoblastic leukemia. However, it is often associated with severe toxic side effects despite its impressive therapeutic efficacy. Sun *et al.* reported that SLC1A3, a transporter protein responsible for the transport of aspartic acid and glutamate, is a potential mediator of ASNase resistance in tumor cells^[147]^. This protein can counteract ASNase-induced depletion of aspartic acid and glutamate, conferring resistance to the cytotoxic effects of the drug.

Furthermore, research has indicated that amino acids, such as asparagine^[148-150]^, leucine^[151]^, isoleucine^[152]^, valine^[153-155]^, and serine^[156-158]^, modulate tumor sensitivity to chemotherapeutic agents. In summary, amino acids not only sustain cancer cell survival by regulating redox homeostasis and promoting anabolic pathways but also assist cancer cells in adapting to therapeutic stress by influencing epigenetic modifications and providing metabolic intermediates generated through energy-producing processes. Consequently, an in-depth understanding of the mechanisms underlying amino acid metabolism related to treatment resistance could provide a foundational molecular basis for the design of more efficacious antineoplastic treatment strategies.

## SEVS REGULATE THE ANTITUMOR THERAPEUTIC RESPONSE THROUGH METABOLIC REPROGRAMMING

Tumor cell metabolic reprogramming involves a diverse array of regulatory molecules, such as transporters, pivotal enzymes, signaling cascades, and oncogenic products. sEVs, which are pivotal conduits for intercellular communication, have been demonstrated to contain molecular constituents implicated in metabolic reprogramming. The role of these vesicles in modulating tumor sensitivity to therapeutic intervention remains elusive, and this role could be a potential regulatory mechanism that significantly impacts the responsiveness of cancers to treatment [Table 1].

**Table 1:** sEVs cargoes involved in cancer cell metabolism and chemoresistance

**Cargo type**	**sEVs cargo**	**Cancer Type**	**Donor cells**	**Recipient cells**	**Biological behavior**	**Ref.**
Protein	Hsp70	Breast cancer	Adriamycin-resistant MCF-7	Adriamycin-sensitive MCF-7	Inhibited respiration, promoted glycolysis, and enhanced adriamycin resistance	[159]
	PKM2	NSCLC	A549	A549	Enhanced glycolytic flux and cisplatin resistance	[10]
	PKM2	Glioma	Hypoxic temozolomide-resistant U251	Sensitive U251, TAMs	Promoted glycolysis and temozolomide resistance	[162]
	PKM2	NSCLC	Hypoxic cisplatin-resistance A549	Sensitive A549, CAFs	Promoted glycolysis and cisplatin resistance	[163]
	TPI, PGK, ENO, PKM, LDHA	Ovarian cancer	Hypoxic CAOV-3	Normoxic CAOV-3	Promoted glycolysis and carboplatin resistance	[164]
	ALDOA, ALDH3A1	Lung cancer	Irradiated A549	A549	Promoted glycolysis	[165]
	LMP1	Nasopharyngeal carcinoma	CNEI-LMP1 (a stable LMP1-integrated cell line)	Fibroblasts, HK1	Promoted glycolysis in CAFs, inhibited glycolysis and promoted OXPHOS in tumor cells	[166]
	ITGB4	Breast cancer	MDA-MB-231	CAFs	Promoted glycolysis in CAFs	[167]
	PD-L1	NSCLC	LLC	Macrophages	Promoted glycolysis and inhibited OXPHOS	[168]
	MTTTP	Colorectal cancer	Adipocytes	SW480, HCT116	Reduced ferroptosis, and promoted chemoresistance to oxaliplatin	[172]
	ACADM	Pancreatic cancer	Pancreatic cancer cell lines	NA	Reduced ferroptosis, chemoresistance to gemcitabine	[173]
	YAP1	Prostate cancer	EnzaR	LNCaP	Promoted lipid metabolism and enzalutamide resistance	[174]
	GSTP1	Breast cancer	Adriamycin-resistant MCF-7	Chemosensitive MCF-7	Promoted glutamine metabolism and adriamycin resistance	[175]
	GLS1	Gastric cancer	Trastuzumab‐resistant NCI‐N87 and trastuzumab‐resistant SNU216	Macrophages	Promoted glutamine metabolism, and trastuzumab resistance	[176]
miRNA	miR-522	Gastric cancer	CAFs	SGC7901, MKN45	Inhibited lipid metabolism and ferroptosis and promoted cisplatin and paclitaxel resistance	[179]
	miR-21-5p, miR-23a-3pand miR-125b-5p	Lung cancer	Paclitaxel-resistant A549	Chemosensitive A549	Promoted unsaturated FA synthesis and paclitaxel resistance	[180]
	miR-3173-5p	Pancreatic cancer	CAFs	PANC-1,BXPC-3	Inhibited ferroptosis and promoted gemcitabine resistance	[178]
	miR-21-3p, miR-21-5p and miR-891-5p	Ovarian cancer	Ovarian cancer cell line	NA	Promoted glycolysis and carboplatin resistance	[181]
	miR-21-5p	Ovarian cancer	Cisplatin-resistant SKOV3	Cisplatin-sensitive SKOV3	Promoted glycolysis and cisplatin resistance	[182]
	miR-3679-5p	Lung cancer	M2 macrophage	A594	Promoted glycolysis and cisplatin resistance	[183]
LncRNA	HISLA	Breast cancer	TAMs	MDA-MB-231	Promoted glycolysis and resistance to docetaxel	[184]
	SNHG3	Breast cancer	CAFs	MCF-7, MD-MBA-453	Promoted glycolysis and inhibited OXPHOS	[185]
	LncFERO	Gastric cancer	SGC7901, MKN45	SGC-CSC, MKN-CSC	Inhibited ferroptosis and promoted cisplatin resistance	[186]
CircRNA	Circ_0094343	Colorectal cancer	NCM460	HCT116	Inhibited glycolysis and improved sensitivity to 5-fluorouracil, oxaliplatin, and doxorubicin	[187]
	Circ_0008928	NSCLC	NA	NA	Promoted glycolysis and cisplatin resistance	[188]
	Circ_0002130	NSCLC	NA	NA	Increased glucose uptake, glycolysis and osimertinib resistance	[189]
	Circ_0005963	Colorectal cancer	Oxaliplatin-resistant SW480	SW480	Enhanced glycolysis and oxaliplatin resistance	[46]
	CircZNF91	Pancreatic cancer	Hypoxic BxPC-3, hypoxic SW1990	Normoxic BxPC-3, normoxic SW1990	Promoted glycolysis and gemcitabine tolerance	[190]
	CircDLGAP4	Neuroblastoma	Doxorubicin -resistant neuroblastoma cells	Doxorubicin -sensitive neuroblastoma cells	Promoted glycolysis and doxorubicin resistance	[191]
mRNA	VEGF/VEGFR mRNA	Acute myeloid leukemia	HL-60, U937	HUVECs	Promoted glycolysis and arabinoside cytopyrimidin resistance	[192]
Phosphorylated signaling protein	p-ERK, p-AKT	Colorectal cancer	LoVo, HCT116	HSC	Promoted lactate metabolism and irinotecan resistance	[193]
Lipid	Acid sphingomyelinase	Multiple myeloma	Drug-resistant U266	Chemosensitive JJN3	Promoted sphingolipid metabolism and melphalan and bortezomib resistance	[11]

sEVs: Small extracellular vesicles; PKM2: pyruvate kinase M2; NSCLC: non-small cell lung cancer; TAMs: tumor-associated macrophages; CAFs: cancer-associated fibroblasts; PGK: phosphoglycerate kinase; LDHA: lactate dehydrogenase A; HK1: hexokinase 1; OXPHOS: oxidative phosphorylation; CSC: cancer stem cell; miRNA: microRNA; lncRNA: long noncoding RNA; HISLA: HIF1α-stabilizing LncRNA; circRNA: circular RNA; mRNA: messenger RNA; VEGF: vascular endothelial growth factor; VEGFR: vascular endothelial growth factor receptor; HUVECs: human umbilical vein endothelial cells; HSC: hematopoietic stem cell.

### Exosomal proteins

Proteins are the primary constituents of exosomes and play a pivotal role in modulating the glycolytic activity of tumor cells. Chemotherapy-resistant cells can confer resistance to chemosensitive cells by transferring exosomes containing Hsp70, which impairs mitochondrial function and increases glycolysis^[159]^. Complementary investigations have revealed a marked upregulation of pyruvate kinase M2 (PKM2) expression in exosomes derived from drug-resistant tumor cells. Elevated PKM2 expression increases glucose uptake and lactate production, contributing to chemotherapy resistance in tumor cells^[10,160,161]^. Notably, the increased expression of PKM2 within exosomes not only causes drug resistance in chemosensitive tumor cells but also influences macrophages and cancer-associated fibroblasts within the tumor immune microenvironment. This phenomenon has significant implications for the development of therapeutic strategies and furthers our understanding of immune responses^[162,163]^. Furthermore, hypoxia-induced PKM2 in exosomes has been suggested to inhibit tumor cell apoptosis, a process contingent upon the PKM2/BCL2 axis^[163]^. In another study, hypoxic conditions were found to increase resistance to carboplatin in ovarian cancer cell lines, an effect associated with the metabolic reprogramming of ovarian cells toward the glycolysis and FA synthesis pathways. Consistent with this finding, exosomes isolated from hypoxia-stimulated OvCar cell lines, as well as plasma from patients with recurrent ovarian cancer, display significantly increased expression of glycolysis-related enzymes^[164]^. Additionally, the role of exosomal metabolic enzymes, such as ALDOA, ALDH3A1^[165]^, LMP1^[166]^, ITGB4^[167]^, and PD-L1^[168]^, in mediating cancer treatment resistance through glycolysis regulation has been extensively documented.

sEVs are instrumental in modulating lipid metabolism, which is pivotal for augmenting tumor sensitivity to therapeutic interventions. Exosomes originating from adipocytes can be internalized by tumor cells, thereby fostering cancer cell proliferation and migration^[169]^. Lazar *et al.* discovered that proteins associated with FAO are packaged within exosomes derived from adipocytes^[170]^. Upon uptake by melanoma cells, these exosomes enhance lipid metabolism in the cancer cells, facilitating tumor invasion and metastasis. Additionally, research indicates that extracellular vesicles from adipose tissue aid in the transport and oxidation of FAs in cancer cells by supplying necessary enzymes and substrates, consequently reprogramming the lipid metabolism of these cells^[171]^. Zhang *et al.* reported that sEVs originating from adipocytes contain elevated levels of microsomal triglyceride transfer protein^[172]^. Moreover, microsomal triglyceride transfer protein expression in colorectal cancer cells was significantly correlated with ferroptosis and sensitivity to the antineoplastic agent oxaliplatin. These effects are due to the inhibition of polyunsaturated FAs and the regulation of lipid ROS levels. Additionally, variations in ferroptosis and ROS levels were noted in exosomes from pancreatic cancer cells with different sensitivities to gemcitabine. The presence of acyl-CoA dehydrogenase medium chains in exosomes was shown to increase the consumption of unsaturated FAs, thereby affecting ferroptosis via the modulation of the glutathione peroxidase 4 and mevalonate pathways^[173]^. Lee *et al.* reported the presence of YAP1 in enzalutamide-resistant cell lines and in sEVs isolated from patient serum^[174]^. Their study underscored the role of YAP1 in regulating genes associated with cancer stemness and lipid metabolism. Notably, enzalutamide-resistant cell lines derived from parent cells treated with sEVs present increased tumorigenic potential, lipid metabolic activity, and robust resistance to enzalutamide.

Exosomes contribute to chemotherapy resistance by transporting key enzymes involved in amino acid metabolism. Yang *et al.* reported that the levels of glutathione S-transferase P1 (GSTP1) within exosomes were significantly greater in Adriamycin-resistant breast cancer cells than in their chemosensitive counterparts^[175]^. Apoptosis assays and immunofluorescence staining of clinical samples from patients undergoing neoadjuvant chemotherapy revealed that GSTP1 expression was markedly greater in patients with progressive disease (PD) or stable disease (SD) than in those who achieved partial response (PR) or complete response (CR). Correspondingly, the serum exosome levels of GSTP1 were also substantially greater in the PD/SD cohort than in the PR/CR cohort, indicating a potential role for exosomal GSTP1 in modulating tumor responsiveness to chemotherapeutic agents. Moreover, Hu *et al.* reported that gastric cancer cells could increase glutamine metabolism by releasing microvesicles enriched with GLS1^[176]^. This process was shown to influence M2 macrophage polarization and angiogenesis within the TME, culminating in the acquisition of trastuzumab resistance in HER2-positive gastric cancer cells.

### Exosomal noncoding RNAs

#### miRNAs

miRNAs are a class of noncoding RNAs that are typically 20-22 nucleotides in length and modulate gene expression posttranscriptionally by binding specifically to the mRNA sequences of target proteins, leading to mRNA degradation or translational inhibition^[177]^. Exosomes derived from cancer-associated fibroblasts encapsulate miR-3173-5p and miR-522, subsequently transferring them to tumor cells. These miRNAs have been shown to suppress iron-dependent cell death mediated by lipid peroxidation in tumor cells, thereby conferring chemoresistance^[178,179]^. Additionally, exosomal miRNAs, such as miR-21-5p, miR-23a-3p, and miR-125b-5p, have been found to inhibit FA synthesis by modulating the TGFβ/SMAD2 pathway, thereby sensitizing tumor cells to paclitaxel therapy^[180]^. In two separate studies, miR-21-5p was demonstrated to activate glycolysis and upregulate drug transporters and detoxification enzymes, contributing to chemotherapy drug resistance^[181,182]^. Notably, miR-21-5p and miR-891-5p have also been implicated in the upregulation of proteins involved in DNA repair mechanisms^[181]^. Wang *et al.* reported that exosomes released by M2 macrophages increase the resistance of lung cancer cells to cisplatin through the transfer of miR-3679-5p. The mechanism underlying this effect involves miR-3679-5p promoting c-Myc protein stability and augmenting glycolysis by downregulating the E3 ubiquitin ligase NEDD4 analog NEDD4L^[183]^.

#### lncRNAs

lncRNAs exceed 200 nucleotides in length and are crucial for modulating tumor responsiveness to therapeutic interventions. sEVs secreted by breast cancer-associated tumor macrophages (TAMs) transfer a specific lncRNA, HIF1α-stabilizing lncRNA (HISLA), to breast cancer cells. This transfer facilitates the stabilization of HIF1α, thereby increasing aerobic glycolysis. This increase results in antiapoptotic effects and fosters chemotherapy resistance in tumor cells^[184]^. Furthermore, Chen *et al.* reported that lactic acid, a glycolytic end-product, upregulates HISLA expression within macrophages, revealing the dynamic relationships and communication between TAMs and tumor cells within the TME^[184]^. Clinically, HISLA expression in TAMs was significantly correlated with therapeutic response and overall survival among breast cancer patients, indicating that HISLA is a potential prognostic biomarker and a valuable adjunct for guiding therapeutic decision-making. Related findings have demonstrated that tumor-associated fibroblasts (CAFs) engage in crosstalk with breast cancer cells via the secretion of exosomes, which promote glycolytic metabolism and proliferation in tumor cells. Mechanistic insights revealed that the lncRNA SNHG3 sequesters miR-330-5p, thereby modulating mitochondrial OXPHOS and glycolysis through the targeted regulation of the pyruvate kinase PKM1/2^[185]^. Additionally, Zhang *et al.* reported that lncFERO, which originates from gastric cancer cells, promotes lipid metabolism and ferroptosis in gastric CSCs via the hnRNPA1/SCD1 signaling axis^[186]^. Both *in vitro* and *in vivo* evidence confirm that chemotherapeutic agents increase the packaging of lncFERO into exosomes and its subsequent release into the extracellular milieu by upregulating hnRNPA1 expression. This process increases the desiccation tolerance of gastric cancer cells, resulting in increased resistance to chemotherapy.

#### circRNAs

circRNAs encapsulated within exosomes have been implicated in the regulation of immune evasion and the progression of malignant tumors through their involvement in metabolic regulation. Li *et al.* reported that exosome-derived circ_0094343 modulates glycolysis via the miR-766-5p/TRIM67 axis, thereby increasing the chemosensitivity of tumor cells^[187]^. In another study, the expression of circ_0008928 in serum exosomes was significantly elevated in patients with cisplatin-resistant NSCLC. Further investigations revealed that circ_0008928 increases glycolysis and decreases cisplatin sensitivity through the miR-488/HK2 signaling pathway^[188]^. Ma *et al.* reported that the expression levels of circ_0002130 were markedly increased in the serum exosomes of patients with osimertinib-resistant NSCLC^[189]^. The underlying mechanism involves circ_0002130 increasing the expression of GLUT1, HK2, and LDHA by sponging miR-498. These factors are all associated with glucose metabolism, leading to increases in glucose uptake, lactate production, and the extracellular acidification rate, indicating increased glycolysis. Thus, circ_0002130 influences osimertinib sensitivity by modulating tumor cell glycolysis, suggesting its potential as a target to impact the drug response. Additionally, circ_0005963 has been shown to increase glycolysis and ATP production in oxaliplatin-resistant cells via the miR-122/PKM2 signaling axis. This process not only increases tumor cell survival but also promotes the transfer of chemotherapy resistance to cells that are otherwise sensitive to chemotherapeutic agents^[46]^. Exosomes derived from hypoxic pancreatic cancer cells increase glycolysis and chemical tolerance in normoxic cells by delivering circZNF91, which functions as a miR-23b-3p sponge. Thus, SIRT1 expression is upregulated, and the HIF-1α protein is stabilized. The presence of circ_ZNF91 in exosomes enables signaling between tumor cells under hypoxic and normoxic conditions, thereby promoting resistance to gemcitabine chemotherapy in pancreatic cancer. The mechanism involves circ_ZNF91 increasing both the transcriptional activity and stability of HIF-1α, resulting in increased glycolysis in recipient pancreatic cancer cells and, consequently, resistance to gemcitabine chemotherapy^[190]^. Furthermore, circDLGAP4, which is carried by sEVs, plays a significant role in neuroblastoma chemotolerance. The authors of this study posit that circDLGAP4 promotes glycolysis and doxorubicin resistance in tumor cells via the miR-143/HK2 axis^[191]^.

### Other regulators affecting metabolism in exosomes

Regulators of sEVs-mediated metabolic reprogramming include mRNAs and phosphorylated signaling proteins. For example, sEVs derived from acute myeloid leukemia cells have been shown to increase glycolysis in human umbilical vein endothelial cells (HUVECs) and increase the expression of vascular endothelial growth factor receptors (VEGFRs). This results in vascular remodeling and causes therapeutic resistance in tumors. The underlying mechanism for these effects is the transfer of VEGF and VEGFR mRNAs via sEVs^[192]^. Another study demonstrated that under normoxic conditions, exosomes from colorectal tumors stimulate interleukin-6 (IL-6) secretion by hepatic stellate cells within the metastatic liver microenvironment through the activation of p-ERK and p-AKT. IL-6, in turn, upregulates the expression of MCT1 and LDHB, which promote lactic acid metabolism in adjacent tumor cells under hypoxic conditions. This culminates in the development of chemotherapy resistance in tumor cells^[193]^. Elevated lactate levels within tumor tissue trigger the MRE11 lactylation, facilitating DNA damage repair and bolstering cancer cells’ resilience to chemotherapy^[194]^. Additionally, the expression of acidic sphingomyelinase (ASM) was significantly increased in multiple myeloma cell lines following treatment with melphalan or bortezomib, as well as in the exosomes they released. Experimental evidence suggests that ASM-enriched exosomes confer drug resistance to chemosensitive cells, highlighting the potential role of ASM in tumor defense mechanisms^[11]^.

In summary, the protein and noncoding RNA contents of exosomes significantly influence the reprogramming of glycolysis, FA metabolism, and amino acid metabolism in tumor cells. Exosomes facilitate intercellular communication, enabling the transfer of drug-resistant phenotypes among cells. Elucidating the specific mechanisms underlying these processes will lay the groundwork for targeted tumor therapy, chemotherapy sensitization, and the utilization of exosomes as diagnostic and prognostic tools.

## CLINICAL APPLICATION OF EXOSOMES TARGETING METABOLIC REPROGRAMMING IN TUMORS

### Diagnostic biomarkers

An increasing body of research has indicated the considerable potential of sEVs in oncological diagnostics, prognostic evaluation, and monitoring of treatment efficacy. In patients with NSCLC, the expression level of circ_ARHGAP10 in serum-derived exosomes is markedly elevated compared with that in healthy control individuals. The upregulation of this molecule is correlated with increased expression of GLUT1 and LDH, both of which are pivotal modulators of glycolysis. Consequently, increased expression of circ_ARHGAP10 may influence tumor energy metabolism and the TME by increasing both the expression of these proteins and glycolytic activity^[195]^. Tang *et al.* reported that the expression levels of six pivotal glycolytic enzymes were significantly increased in salivary exosomes from patients with HPV-linked oropharyngeal cancer^[196]^. These enzymes include ALDOA, GAPDH, LDHA/LDHB, PGK1, and PKM1/2. This discovery reveals a novel role for salivary exosomes in modulating the interplay between glucose metabolism and HPV-driven oropharyngeal cancer and suggests their utility as biomarkers for the diagnosis of this disease. Additionally, exosome contents related to glycolysis, such as PKM2 and circPDK1, are also considered potentially valuable for cancer diagnosis^[196,197]^.

Extensive alterations in the lipid composition of exosomes originating from cancer cells have been reported, indicating their potential utility as biomarkers for cancer screening^[198]^. For example, lipid metabolism in exosomes derived from HCC patients is markedly distinct from that in exosomes derived from non-liver cancer patients. Specifically, there is a significant increase in the levels of lipid molecules, such as sphingosine, diacylglycerol, lysophosphatidic acid, and (O-acyl)-1-hydroxy FA, whereas the levels of sulfatides and acylGlcSitosterol esters are reduced^[199]^. Tao *et al.* reported that, compared with those in healthy individuals, serum-derived exosomes in pancreatic cancer patients exhibit substantial lipidomic shifts involving 20 lipid species^[200]^. Notably, the levels of certain lipid species, including LysoPC (22:0), phosphatidylcholine (PC) (P-14:0/22:2), and phosphatidylethanolamine (PE) (16:0/18:1), were significantly correlated with clinical tumor stage, the levels of the tumor markers CA19-9 and CA242, and tumor diameter. Notably, the level of PE (16:0/18:1) was also significantly associated with overall survival. Furthermore, the glycerophospholipid choline PC (16:0/0:0) is expressed at significantly higher levels in exosomes from melanoma CSCs than in those from their differentiated counterparts, positioning it as a potential biomarker for melanoma diagnosis^[201]^. These findings underscore a potential relationship between perturbations in lipid metabolism within cancer patient-derived exosomes and disease progression, suggesting that these lipid species could serve as promising biomarkers for early-stage tumor detection.

### Prognostic biomarkers

As previously reported, the expression of exosomal HISLA is closely related to tumor glycolysis. Moreover, elevated HISLA expression serves as a valuable indicator for assessing tumor histological grade, clinical stage, lymph node metastasis, and HER2 subtypes. Specifically, in breast cancer patients who exhibit PD or SD during treatment, HISLA expression levels were markedly greater than those observed in patients who achieved partial or complete remission^[184]^. Furthermore, an investigation revealed a significant enrichment of glycolytic pathway proteins within exosomes secreted by ovarian cancer cells under hypoxic conditions. These findings suggest that these proteins could predict ovarian cancer recurrence in clinical settings^[164]^.

Qi *et al.* reported that the expression level of miR-3173-5p in exosomes was significantly greater in PDAC tissues than in adjacent normal tissues, concomitant with the significant suppression of its presumptive target gene ACSL4^[178]^. In PDAC patients undergoing chemotherapy, the expression of miR-3173-5p in tumor tissues was notably increased posttreatment compared with pretreatment levels. These observations implicate miR-3173-5p in the promotion of cancer malignancy. Additionally, ACSL4 expression has been recognized as an effective predictor of 5-year survival in pancreatic cancer patients. In another study, piRNA-17560, which is present in exosomes derived from senescent neutrophils, increased the expression of obesity-associated proteins, thereby inducing resistance in breast cancer cells to the chemotherapy agent docetaxel. Notably, plasma levels of piR-17560 are significantly greater in patients who exhibit poor responses to chemotherapy than in those who exhibit favorable responses^[202]^. These findings suggest that exosomes may be promising diagnostic and prognostic biomarkers for cancer therapy by targeting metabolic reprogramming.

### Exosomes as therapeutic targets

To combat drug resistance mediated by sEVs, two primary strategies can be employed to increase the efficacy of chemotherapy: (1) diminishing their concentration within the TME by inhibiting the biogenesis and release of exosomes; and (2) neutralizing the resistance-promoting molecules, such as RNAs, proteins, or metabolites, carried by exosomes, thereby undermining their protective influence on tumor cells. In this section, we focus on targeting exosomal contents to modulate tumor metabolism and therapeutic resistance.

Exosomes have garnered significant attention as potential therapeutic targets in the field of tumor therapy. Pan *et al.* reported that the lncRNA IGFL2-AS1 could promote sunitinib resistance by regulating autophagy^[203]^. Using a patient-derived xenograft model of sunitinib-refractory metastatic renal cell carcinoma, the authors demonstrated that the delivery of antisense oligonucleotides against IGFL2-AS1 via chitosan-coated solid lipid nanoparticles effectively reversed drug resistance. As previously discussed, exosomes play a pivotal role in metabolic reprogramming within tumors. We hypothesize that targeting specific regulatory mechanisms could impede cancer progression by modulating metabolic pathways^[185,191,192,204]^. For example, circ_0005963 induces oxaliplatin resistance in colorectal cancer cells by activating the miR-122/PKM2 signaling axis, thereby promoting glycolysis and ATP production. Further studies revealed that exosomes containing si-circ_0005963 could be efficiently produced by transfecting small interfering RNAs into HEK293T cells. Treatment with exo-si-circ_0005963 counteracted oxaliplatin resistance in CRC cells *in vitro* by inhibiting the aforementioned pathways^[46]^. circCCT3 is upregulated in clinical HCC tissues and influences glucose metabolism in HCC cells by regulating HK2. Coptisine can inhibit the expression of circCCT3 in CAF exosomes, thereby inhibiting the malignant progression of HCC^[205]^. Another study revealed that TAMs mediate aerobic glycolysis and chemoresistance in tumor cells via lncRNAs that shuttle HIF1α to breast cancer cells. Targeting the silencing of HISLA in TAMs to abrogate secretory HISLA in sEVs significantly impedes the ability of sEVs to induce resistance to apoptosis in tumor cells under chemotherapy^[184]^.

Exosomes can render tumor cells less susceptible to treatment by inhibiting ferroptosis induced by lipid peroxidation. Du *et al.* successfully encapsulated the ferroptosis inducer erastin and the photosensitizer Rose Bengal into exosomes via ultrasonic technology^[206]^. The engineered drug-carrying exosomes (Er/RB@ExosCD47) generated through this approach potently induced ferroptosis in tumor cells upon laser activation at a wavelength of 532 nm both *in vitro* and *in vivo*.

Importantly, exosomes play a significant role in tumor progression, positioning them as viable therapeutic targets with multiple advantages. Despite numerous *in vitro* investigations exploring the use of exosomes in cancer treatment, clinical trials remain scarce. We postulate that a targeted therapeutic approach aimed at impeding exosome production and metastasis could potently hinder tumor progression, particularly metastasis. The concurrent targeting of exosomes and cancer cells has led to promising outcomes in combating cancer progression, suggesting a potential future therapeutic strategy for tumor management.

### Engineered exosomes for drug delivery

sEVs are not only a research hotspot in the field of tumor diagnosis and treatment but also show significant potential in the field of targeted drug delivery. These nanoscale particles have unique biological properties. With diameters of less than 200 nm, they are capable of crossing the blood-brain barrier. Their lipid bilayer membranes and internal space allow for the encapsulation of molecules or drugs, and they demonstrate very low immunogenicity. Their surface can be modified by physical or chemical methods and can be effectively endocytosed by target cells, thereby mediating intracellular signaling. Owing to these advantages, exosomes have received much attention as drug delivery platforms that regulate cellular metabolism and are considered promising drug carriers. Studies have shown that sEVs functionalized with hyaluronic acid (HA) can efficiently deliver doxorubicin to drug-resistant breast cancer cells as drug carriers. This specific cancer-targeting ability is achieved through a mechanism mediated by the CD44 receptor. In addition, in preclinical multidrug-resistant tumor models, HA functionalized sEVs (lipHA-hsEVs) effectively inhibited local tumor growth and significantly reduced the systemic toxicity of DOX^[207]^. Moreover, Lin *et al.* found that CPT1A, a key regulatory enzyme in the FAO pathway, was significantly highly expressed in oxaliplatin-resistant colon cancer cell lines^[208]^. Further studies revealed that pharmacological inhibition of CPT1A activity effectively reversed oxaliplatin resistance in these cells and promoted apoptosis. The specific delivery of siCPT1A to tumor tissues can be achieved by using exosomes modified with the iRGD peptide as drug carriers. This strategy successfully restored the sensitivity of colon cancer cells to oxaliplatin by inhibiting the activity of the FAO pathway, providing an innovative targeted therapy to solve the problem of chemotherapy resistance.

## CONCLUSION

Given their potential as diagnostic biomarkers and therapeutic agents, sEVs have become a focal point in cancer research, especially in the context of their application as a drug delivery platform. Prospective research endeavors should concentrate on addressing four critical inquiries. (1) How can metabolic reprogramming facilitated by sEVs be fully harnessed to establish novel clinical therapeutic pathways? (2) How can the targeting efficacy of engineered sEVs be enhanced to reverse metabolic alterations in recipient cells and circumvent the evolution of chemotherapy resistance? (3) How can the efficiency of sEVs as drug transporters be optimized to reduce costs and increase therapeutic outcomes? (4) While recent investigations are predominantly anchored in *in vitro* cellular assays and animal models, translation into clinical studies remains scarce, chiefly owing to financial and ethical constraints. Despite these challenges, pioneering research on sEVs-mediated metabolic reprogramming is paving the way for groundbreaking advancements in future cancer therapeutics.

## DECLARATIONS

### Copyright

© The Author(s) 2024.
